# The research rotation: competency-based structured and novel approach to research training of internal medicine residents

**DOI:** 10.1186/1472-6920-6-52

**Published:** 2006-10-17

**Authors:** Balavenkatesh Kanna, Changchun Deng, Savil N Erickson, Jose A Valerio, Vihren Dimitrov, Anita Soni

**Affiliations:** 1 Department of Internal Medicine, Lincoln Medical & Mental Health Center, New York, USA, Affiliated with Weill Medical College of Cornell University, New York , USA; 2 Research Assistant Program of the Graduate Medical Education Office, Lincoln Medical & Mental Health Center, New York, USA, Affiliated with Weill Medical College of Cornell University, New York, USA

## Abstract

**Background:**

In the United States, the Accreditation Council of graduate medical education (ACGME) requires all accredited Internal medicine residency training programs to facilitate resident scholarly activities. However, clinical experience and medical education still remain the main focus of graduate medical education in many Internal Medicine (IM) residency-training programs. Left to design the structure, process and outcome evaluation of the ACGME research requirement, residency-training programs are faced with numerous barriers. Many residency programs report having been cited by the ACGME residency review committee in IM for lack of scholarly activity by residents.

**Methods:**

We would like to share our experience at Lincoln Hospital, an affiliate of Weill Medical College Cornell University New York, in designing and implementing a successful structured research curriculum based on ACGME competencies taught during a dedicated "research rotation".

**Results:**

Since the inception of the research rotation in 2004, participation of our residents among scholarly activities has substantially increased. Our residents increasingly believe and appreciate that research is an integral component of residency training and essential for practice of medicine.

**Conclusion:**

Internal medicine residents' outlook in research can be significantly improved using a research curriculum offered through a structured and dedicated research rotation. This is exemplified by the improvement noted in resident satisfaction, their participation in scholarly activities and resident research outcomes since the inception of the research rotation in our internal medicine training program.

## Background

Graduate medical education in the field of Internal Medicine (IM) in the United States consists of mainly clinical experience and medical education while emphasis on research training is often inadequate or non-existent. The Accreditation Council of graduate medical education (ACGME) requires all accredited Internal medicine residency training programs to ensure that residents participate in scholarly activities as follows: Each program must provide an opportunity for residents to participate in research or other scholarly activities, and residents must participate actively in such scholarly activities [1].

In the effort to comply with these requirements, IM residency training programs are left to design the research structure, process and outcome evaluation. In light of varying resources available to the programs, it is not feasible to maintain uniform standards in meeting the research requirement. Inadequate demonstration of residents' scholarly activities is one of the most frequent citations by the ACGME. About 10% of residency programs report having been cited by the residency review committee in IM for lack of scholarly activity by residents, which places these programs at risk for a shortened accreditation cycle [2].

Residents' research lead to better clinical care, [3] correlates with the pursuit of academic careers, [4] increases numbers of clinician investigators, [5] and is an asset to those applying for fellowships [6]. The National Graduate Medical Education Census [7] reported 101,291 residents and fellows enrolled in ACGME-accredited and combined specialty GME programs for the 2004–2005 academic year. Of these, 21332 were internal medicine residents (22%). Active and meaningful involvement in research during residency training will lay the foundations and provide the skills needed for life long learning expected from all internal medicine doctors.

Most residents and faculty find it difficult to conduct research because of other priorities such as patient care. Additional constraints include lack of financial support, lack of research resources such as libraries and computers and online medical databases, lack of interest, and lack of mentoring and support staff with expertise in statistics. Non-university programs in general encounter more difficulties in terms of resources [8].

We report our success in designing and implementing a structured research curriculum incorporating basic principles within a research rotation to enhance participation and outcomes of our residents in scholarly activities within a busy residency training program setting.

## Methods

### Program Description

Lincoln Medical & Mental health Center is a busy city hospital affiliated with Weill Medical College of Cornell University serving the population of the South Bronx in New York City. It is part of the New York City Health and Hospital Corporation, the public hospital system of New York City. 120 of the total 342 beds are in internal medicine, with approximately 11000 annual inpatient medical discharges and 100,000 outpatient visits. The Department of Internal Medicine consists of 54-fulltime board certified faculty in Internal Medicine and subspecialties. The total number of residents in the categorical track of IM residency training program is 84.

### Needs assessment & Research Curriculum implementation

The needs assessment for a structured approach to research training was based on the annual on-line ACGME IM resident program survey in the year 2004. According to this survey, our residents felt that opportunities for participation in scholarly activities were insufficient. In view of the results, we re-evaluated research training in our program utilizing the Avedis Donabedian health care improvement model of structure, process and outcomes [9]. A detailed survey was administered to understand specific needs of residents. Based on our findings, we designed a structured research curriculum with goals and objectives incorporating ACGME competencies. The following structural components and processes were initiated or strengthened if already pre-existing.

### Structure elements

• Dedicated Faculty: A qualified faculty member with masters of public health was appointed as research director to plan and oversee the implementation of a structured approach to research training. The research director was allowed protected time to administer, teach and evaluate all research components.

• On-line resources: Computer and software programs required for literature searches and on-line databases were made available to all residents and faculty with 24/7 access from any computer terminal.

• Research rotation: refer section below

### Process elements

• Core lecture series on biostatistics and research methodology: An intensive 2-hour weekly sessions on research methodology is conducted for 4 weeks every year for all residents by qualified public health and statistics faculty from the university affiliation. During these sessions, fundamental concepts in biostatistics and research designs are discussed. In addition, residents are provided dedicated time to attend didactic lectures in an annual core curriculum series of lectures on basic biostatistics, how to do research, search strategies and critical appraisal of literature.

• Opportunities for presentation of research projects: An annual Research competition is conducted by the department, where residents are invited to present posters and individual prizes are awarded for best research posters, evidenced based topic reviews and clinical vignettes. The research director conducts periodic research-in-progress seminars where residents are provided an opportunity to discuss their research projects among themselves.

• Program research requirement for residents: All residents are required to participate and complete at least one scholarly activity during their training period under guidance of a faculty mentor.

• Communication of research outcomes: All research projects completed by residents and presented at regional, national conferences or published are communicated to all program residents and faculty through a poster board as well as in monthly resident meetings by the research director.

### Research rotation

The highlight of our re-vitalized approach to structured research training is the research rotation. The research rotation was designed specifically to enhance research among residents and also meet ACGME scholarly activities requirements.

#### i) Goals

Scholarship is defined as the following by ACGME a) the scholarship of discovery, as evidenced by peer-reviewed funding or by publication in a peer-reviewed journal. b) the scholarship of dissemination, as evidenced by review articles or chapters in textbooks. c) the scholarship of application, as evidenced by the publication or presentation at local, regional, or national professional and scientific society meetings. The main goals of the research rotation are to ensure that all Internal Medicine residents are able to:

• Understand search strategies while utilizing online literature databases

• Ascertain limitations in their critical appraisal of literature and means to address those deficiencies.

• Understand limitations and advantages of original peer-reviewed medical literature.

• Discuss and understand sensitivity, specificity and predictive values of diagnostic tests, how they are used, and how tests are selected and interpreted; understand the impact of prevalence of disease on the predictive values (and the interpretation) of a diagnostic test.

• Understand the meaning of statistical significance and differentiate it from clinical significance; learn to evaluate observational study designs such as cross-sectional, case-control, and cohort studies.

• Discuss bias and confounding

• Understand the structure of randomized control trial, its strengths and weaknesses

• Compile evidenced based review of a specific clinical question using critical appraisal skills

• Understand how to design a protocol for an original research project utilizing fundamental statistical principles and research methodology

• Present results of the research activities conducted in an appropriate forum.

#### ii. Research rotation components & evaluation

The following major components were designed to address goals of the research rotation. (Refer Additional file 1 for detailed description of the components)

• Evidenced based topic: Collect literature evidence and draw conclusions regarding a clinical question

• Critique of Literature: Critically appraise articles from medical literature

• Research topics: Understand study designs, basic statistical concepts and interpretation of results

• Diagnostic testing in clinical practice: Understand reliability of diagnostic tests

• Original research project option: Design a new study protocol utilizing the information and knowledge acquired during the rotation.

• Literature search strategy: A dedicated session with a qualified librarian to learn medical database information and search methods.

Each resident spends two weeks in the research rotation i.e. 2 residents within the usual 4-week period rotation schedule. All residents rotate through elective under the supervision of the faculty research director. A pre-selected faculty mentor can also act as project guide. Residents are expected to participate actively in the components of the rotation. Guidelines are provided as handouts for all components of the rotation. The faculty research director meets the resident daily during the research rotation to oversee participation of the resident in the various components as well as discuss topics required for understanding biostatistics and research methodology. A detailed timetable of daily activities is provided at the beginning of the rotation to guide the resident through the different days and components of the rotation (Additional file 2).

The daily schedule includes the following sessions with starting time: Morning report 8:30 AM; Required reading 9:30 AM; Research topic preparation 10:30 AM; Noon conference 12 Noon; Original Research project 1:30 PM; Departmental Project (optional) 3:30PM till 4:30 PM. Residents do not have any clinical responsibilities during the rotation and can give undivided attention to participation and learning of the critical elements of the research rotation.

Based on performance of the resident among the various research rotation components, an ACGME competency based evaluation is provided at the end of the rotation. (Additional file 3) Residents are also asked to submit a research elective evaluation (Table 1).

## Results

### Research rotation outcomes

The research rotation was initiated in May 2004. Every month since then, two medical residents were randomly assigned to the research rotation through a schedule prepared independently by the chief medical resident in consultation with program faculty. Until the beginning of our study, 43 residents have completed this rotation. All others residents will be assigned to the research rotation before the completion of their Internal Medicine training. This will certainly help satisfy the ACGME requirements for research training for our residents.

Our study objective is to compare the effect of this rotation on general and ACGME competencies related to research curriculum among residents who have already completed the research rotation and those who have still not been assigned to the rotation.

We therefore systematically measured the impact of this rotation on resident research activities using the following different measures:

• Resident research rotation evaluation

• General resident research satisfaction and ACGME competency based self assessment surveys

• Publication, presentation & participation in scholarship as defined in the ACGME requirements

#### a) Resident research rotation evaluation

All residents who rotate through the research elective provide feedback regarding their satisfaction and assessment of workload, comprehensiveness, supervision, new learning experience and interest during the rotation. The results of evaluation from 39 of 44 residents who rotated through the research elective since 2004 are shown below. (Table 1)

Most residents felt that the rotation stimulated their interest in research, was comprehensive, provided them a new learning experience in a short period of time and workload was appropriate. The mean scores for all items in the evaluation was above 4 on a scale of 1 to 5, 5 being excellent, reflecting resident satisfaction in learning and participation among research components of the research rotation.

#### b) General resident research satisfaction survey and ACGME competency based self assessment survey of all residents

Since the research rotation evaluation captures the feedback of only those who have rotated in this elective so far, we performed an anonymous and independent resident research satisfaction survey among a random sample of 42 residents in our program recently in order to assess the overall satisfaction. The scores reported in general resident research surveys were also based on a 1 to 5 scale (1 poor 2 satisfactory 3 average 4 good 5 excellent).

In addition, to assess perception of residents among competencies related to scholarly activities, a self administered ACGME competency based evaluation on research training in our program was also obtained from a random sample of residents. The ACGME competency based self evaluation survey was scored from 1 to 10 (1–3 unsatisfactory 4 – 7 satisfactory 7–9 superior) A detailed description of how the components of the research elective administered suits the ACGME competencies is provided in the evaluation from in Additional file 3.

The scores reported by residents who have rotated in research elective thus far (rotators) were compared with those who are yet to undergo this experience (non-rotators). The null hypothesis was that resident research rotation does not have any impact on resident self -perception of knowledge and skills in research. In view of sample size, the median scores were calculated and statistical test of significance for non-parametric Mann Whitney U test or Fischer exact testing was applied. A p value of <0.05 was considered as significant. Results are shown in Table 2 &3.

Fifty residents responded to both the general satisfaction and ACGME competency based self-assessment surveys. Of these, 7 responses to the general survey and 8 in the ACGME self-assessment survey were incomplete. All surveys were conducted by independent observers blinded to the objectives of the survey and therefore did not have any prior knowledge regarding the residents' rotation or training. The reliability of the responses to the items in the questionnaires was addressed by analyzing repeated measurements at different times by independent observers.

According to the general survey, (table 2) all residents believe that research training is very important part of their training. They also felt that more time is needed for research activities. Compared to non-rotators, residents who completed research rotation namely rotators, felt that they were able to utilize available resources better, although the difference was not statistically significant. (p = NS) However, research rotators were able to utilize the expertise of the research director for their scholarly activities and this difference between the two groups is statistically significant. In addition, all research rotators rated higher their skills in the components of research compared to the non-rotators. (p < 0.05 for components 1& 3 of item #4)

In the ACGME competency based self-evaluation, research rotators consistently scored higher in all competency-based elements of research suggesting that the research rotation increased awareness and confidence regarding competencies in research activities. However, the difference was not statistically significant. (p = NS) (Table 3)

#### c) Publication, presentation & participation in scholarly activities as defined in the ACGME requirements

Lastly and most importantly, we analyzed the ACGME defined scholarly activities outcomes among all residents. In order to do this, we collected information on all research, peer reviewed articles, reviews, book chapters, presentations, journal club & other conferences in which residents have participated and significantly contributed since year 2004 when the research rotation was initiated. Results were compared between rotators and non-rotators. As our program's goal is to ensure participation of all residents in scholarly activities, we analyzed the outcomes based on the number of residents who participated in research activities, rather than the number of projects per resident. Using non-parametric Fisher's exact test, the total number of residents with scholarly activities were analyzed between the two groups. A p value of <0.05 was considered significant.

Results of the research outcomes analysis are shown in Table 4. Data was available for 81 residents in the training program. Of these, 43 residents have completed the research rotation. The rotators group had significantly more residents participating in scholarly activities than the non-rotators (p < 0.001). The rotators group also outperformed the non-rotators group in total research projects (published & non-published) (p < 0.001) and letters to editors (p < 0.001). The number of residents who were able to publish among the rotators was marginally significant than the other group (p = 0.053). There were no significant differences in presentations between the two groups.

Of special interest to us is our recent success in publishing letters to the editors to various high quality peer reviewed journals including JAMA, Annals of Internal Medicine, Archives of Internal Medicine, BMC Critical care & European heart Journal. Of the total 16 letters completed, 6 among the rotators group and one in the non-rotators group have been published or accepted for publication in premier internal medicine journals, which highlights the research rotation's remarkable success within a short time. [10-16]

## Discussion

Medical research is an important tool in patient care. Although, it is not reasonable to expect that all residents to be researchers, an Internal Medicine resident is expected to understand literature and critically appraise evidence prior to application in day to day practice. The educational culture among residency training programs tends to be driven by inpatient hospital care with increased emphasis on primary care in the past few years. However, the focus on research has been sub-optimal among residency training programs. Some of barriers to implementation of a structured research curriculum are the lack of specific goals and objectives. Although ACGME requires scholarship, objectives and outcomes to measure programs' efforts in research training are not well defined.

Designing and implementing a structured research rotation involves an increased role for faculty as mentors to residents. It also requires that faculty be provided with dedicated time to oversee the components of the rotation. The supervising faculty must be qualified to teach the research components. A detailed daily work schedule, timetable, specific components and time frame are essential for success of the rotation. Finally the evaluation of residents in research should be based on ACGME competencies in order to standardize the assessment of resident's competency in various components.

Our experience with introducing a research rotation as part of the curriculum has been quite encouraging. Besides the above benefits, participation in research rotation allows us to evaluate our residents in the following core competencies – practice based learning, communication skills, and to a certain extent system based practice. In order to be successful, we have implemented certain rules regarding the rotation. For example, residents involved in research are not involved in patient care and have no clinical responsibilities. The fact that these residents cannot be re-assigned to cover any clinical needs (sick calls, etc), clearly sends the message that the program values this experience and the residents have responded by engaging actively in the research.

## Conclusion

There are several obvious benefits of a structured research rotation as an integral part of a residency training program: Introduction of the housestaff to the basic principles of medical research, provide the knowledge and skills necessary to engage in research activity (formulating a hypothesis, understanding statistical methods and their application, plan and execute the actual research, learn to use the available databases, address research ethics, maintain highest standards of research integrity when collecting and interpreting the data), enforce the principle of evidence-based medicine as a corner-stone for effective medical practice, develop skills necessary to critically analyze and interpret published data and create the foundation for life-long self learning and improvement for the medical practitioners.

Through a structured research rotation, the culture of our residents has changed dramatically since its introduction. Most of our residents appreciate the importance of research for patient care in the day-to-day practice of medicine. This phenomenon is demonstrated by the improvement in resident satisfaction, participation and a significant increase in research outcomes.

## Abbreviations

ACGME Accreditation Council of Graduate Medical Education

IM Internal medicine

NS Not significant

JAMA Journal of American Medical Association

BMC Biomed Central

## Competing interests

The author(s) declare that they have no competing interests.

## Authors' contributions

BK was responsible for design of the concept, research curriculum development, research rotation goals and objectives, directing the research rotation, study & evaluation methods, manuscript preparation and data analysis. CD participated in the study survey data methods, data base and manuscript preparation. SE & JV conducted the study survey methods, data collection, database entry and data cleaning. VD & AS served as advisors for the design of concept, research rotation goals & objectives, evaluation methods, and manuscript preparation. All authors read and approved the final manuscript.

## Pre-publication history

The pre-publication history for this paper can be accessed here:



## Supplementary Material

Additional File 1 Research rotation components. The major components that were designed to address goals of the research rotation Click here for file

Additional File 2 Research elective plan. A detailed timetable of daily activities to guide the resident through the different days and components of the rotation Click here for file

Additional File 3 Research rotation competency based evaluation form. An ACGME competency based evaluation provided at the end of the rotation to residents based on performance of the resident among the various research rotation components Click here for file

## Figures and Tables

**Table 1:** Research rotation evaluation (n = 39)

**Items evaluated**	Mean Scores*	**Range**
Was the amount of work appropriate	4.5	3 – 5
Was the curriculum comprehensive and defined?	4.7	3 – 5
Was the tutor helpful in assisting needs and objectives?	4.8	3 – 5
Was the elective useful in stimulating our interest in scholarly activities?	4.7	3 – 5
Did you develop new skills and interests during the rotation?	4.6	3 – 5
Overall experience	4.7	3 – 5

* 1 = poor; 2 = satisfactory; 3 = average; 4 = good; 5 = excellent

**Table 2:** General resident research survey results (n= 43)

**Response to survey questions**	**Non-rotators (n= 15)**	**Research rotators (n = 28)**	P Value*
	Yes/Total		
**1**. # of residents who believe research is important in residency training?	15	24	NS
**2**. Ever use (where applicable) the following resources for research during your training?			
Research Director	7	27	<0.05
Faculty mentors/assistance	11	24	NS
On line resources (database searches, journals, presentation support – computer, software etc)	13	28	NS
Time (other than research rotation)	9	22	NS
Biostatistics course	11	21	NS
Research requirement for graduation	12	25	NS
Adequate emphasis on research activities by program	12	26	NS
Department research competition	10	15	NS
Other research forum opportunities (Institutional, State, National conferences)	9	18	NS
**3**. What is the single most important barrier to research during the time of residency training?			
• Lack of resident time	14	27	NS
• Lack of resident interest	4	7	NS
• Lack of money	2	5	NS
• Lack of teaching program or materials-	2	4	NS
• Lack of faculty time	6	7	NS
• Lack of computers or software	0	1	NS
• Lack of faculty role models and mentors	6	6	NS
• Lack of faculty interest	4	7	NS
**4**. Residents rating of their specific training in the following components of research during your residency training?			
	Mean Score+		
Evidenced based topic review (e.g. to search articles on a specific clinical question and gather articles, critique, rank evidence and conclude the answer to the clinical question)	3.2	4.1	<0.05#
Critical appraisal of literature (e.g. analyze and interpret individual study findings)	3.4	3.7	NS#
Original research projects	2.9	3.7	<0.05#
Journal club discussions	3.4	3.7	NS#

* by Fisher's exact test for proportions

# by Mann Whitney U test

+ 1 poor 2 satisfactory 3 average 4 good 5 excellent

**Table 3:** *ACGME competency-based self-evaluation (median scores; n = 42)

**#Research Component**	**Competency tested**	**Non-rotators (n= 14)**	**Rotators (n = 28)**
Evidence based topic	Practice based learning	6.25	6.75
Critique of literature	Medical knowledge	5	6
Designing research project	Medical knowledge/Interpersonal skills	6	6.75
Research interest/learning	Professionalism/Medial knowledge	6.5	7
Data gathering and analysis	System based practice/Practice based learning	6.25	7
Overall Competence in research skills	All of the above	6	7

*p value using Mann Whitney U test for comparison of median scores between rotators & non-rotators were non-significant.

# Score 1–3 unsatisfactory; Score 4–7 satisfactory; 7–9 superior

**Table 4:** **Resident research outcomes (n = 81)

**Item**	**Research rotators (n = 43)**	**Non-rotators (n = 38)**	P value*
# of residents with at least one scholarly activity	**40**	**19**	**<0.001**
**Publication**			
# of residents who have at least one projects intended for publication	**33**	**9**	**<0.001**
# of residents with at least one published project	**9**	**2**	**0.053**
# of residents who have at least one project in progress	**23**	**4**	**<0.001**
# of residents with any project that could not be published	**6**	**3**	**NS**
**Category of publication**			
Research	**19**	**7**	**<0.001**
Review	**1**	**1**	**NS**
Case-report	**4**	**1**	**NS**
Letter to the editor	**15**	**1**	**<0.001**
**Presentation or Abstract submission to National/State level conferences**			
# of residents with at least one presentation/abstract	**24**	**17**	**NS**
# of residents who have at least one abstract accepted for presentation	**16**	**11**	**NS**
# of residents who have any abstracts not accepted	**3**	**8**	**NS**
# of residents with at least one abstract preparation in progress	**9**	**2**	**NS**
# of residents who present at national conferences	**5**	**1**	**NS**
# of residents who present at state level conferences	**18**	**16**	**NS**

* Based on Fisher's exact test

**Please note that each resident may have several projects at various stages of completion and in different categories.
