# Supplementary material for: The research rotation: competency-based structured and novel approach to research training of internal medicine residents
Source: BMC Med Educ. 2006 Oct 17;6:52. doi: 10.1186/1472-6920-6-52 (PMC1630691; doi:10.1186/1472-6920-6-52)
Supplement: Additional File 1 — Research rotation components. The major components that were designed to address goals of the research rotation [file 1472-6920-6-52-S1.doc]

# Title:

# The research rotation: competency-based structured and novel approach to research training of internal medicine residents

**Authors:**

Balavenkatesh Kanna^1^ Associate Program Director of Internal Medicine

Assistant Clinical Professor of Medicine

Changchun Deng ^2^

Savil N.Erickson ^3^

Jose A. Valerio^4^

Vihren Dimitrov^5^

Associate Program Director of Internal Medicine

Assistant Clinical Professor of Medicine

Anita Soni^6^

Chair & Program Director of Internal Medicine

Associate Professor of Medicine

**Institutional Affiliation:**

^1,2,5,6^ Department of Internal Medicine, Lincoln Medical & Mental Health Center, New York USA, Affiliated with Weill Medical College of Cornell University, NewYork USA

^3,4^ Research Assistant Program of the Graduate Medical Education Office, Lincoln Medical & Mental Health Center, New York USA, Affiliated with Weill Medical College of Cornell University, NewYork USA

**Corresponding Author:**

Name: Balavenkatesh Kanna MD MPH
Address: 500, Central Park Avenue, Unit # 437, Scarsdale NewYork 10583

Phone: 914-912-8320

Office: 718-579-5000 ext 5016

Fax: 718-579-4836

Email: bvkanna@aol.com

**Additional file 1:**
File format: MS Word
Title: Research rotation components
Description: The following major components were designed to address goals of the research rotation

Evidence based medicine Topic

- Topic name

A topic of your interest in the field of Internal Medicine

Examples of topics chosen: Clinical value of serum BNP; Clinical measures of GFR; Grave’s ophthalmopathy – mechanisms and therapy

- Instructor

A mentor who will be actively involved in this project

- Date of Submission

At the end of the 2 week rotation

Critical Appraisal of literature

- Article topic

Selected major trial from your field of interest

- Instructor

Research Director

- Date of review

During the 2nd week towards the end of the rotation

Present a summary of critiques of the article in written format based on principles of biostatistics - to a peer-reviewed journal if possible

Research elective topics

- Required reading: A list of topics to be learnt and references is provided.

p-value/ Confidence Intervals

Odds ratio & Relative risk

Study designs

Number needed to treat & Number needed to harm

Sensitivity, specificity, PPV, NPV

Measures of central tendency- mean, median, mode

How to critique an article with randomized controlled trial

Bias & Confounding

- Brief afternoon discussions with research director
- Evaluation using multiple choice exam at the end of the rotation

Original research project

- Topic

A topic of your interest in the field of Internal Medicine

Intent to publish in peer reviewed journals or present at scientific meetings

- Instructor/ Faculty Investigator

A faculty member with active interest in the project

- Date of Initiation

Form an idea and start formulating the components of the write-up.

Approximate Completion date: Resident will assign a reasonable period of time to develop and perform the study
