# Supplementary material for: The research rotation: competency-based structured and novel approach to research training of internal medicine residents
Source: BMC Med Educ. 2006 Oct 17;6:52. doi: 10.1186/1472-6920-6-52 (PMC1630691; doi:10.1186/1472-6920-6-52)
Supplement: Additional File 2 — Research elective plan. A detailed timetable of daily activities to guide the resident through the different days and components of the rotation [file 1472-6920-6-52-S2.doc]

# Title:

# The research rotation: competency-based structured and novel approach to research training of internal medicine residents

**Authors:**

Balavenkatesh Kanna^1^ Associate Program Director of Internal Medicine

Assistant Clinical Professor of Medicine

Changchun Deng ^2^

Savil N.Erickson ^3^

Jose A. Valerio^4^

Vihren Dimitrov^5^

Associate Program Director of Internal Medicine

Assistant Clinical Professor of Medicine

Anita Soni^6^

Chair & Program Director of Internal Medicine

Associate Professor of Medicine

**Institutional Affiliation:**

^1,2,5,6^ Department of Internal Medicine, Lincoln Medical & Mental Health Center, New York USA, Affiliated with Weill Medical College of Cornell University, NewYork USA

^3,4^ Research Assistant Program of the Graduate Medical Education Office, Lincoln Medical & Mental Health Center, New York USA, Affiliated with Weill Medical College of Cornell University, NewYork USA

**Corresponding Author:**

Name: Balavenkatesh Kanna MD MPH
Address: 500, Central Park Avenue, Unit # 437, Scarsdale NewYork 10583

Phone: 914-912-8320

Office: 718-579-5000 ext 5016

Fax: 718-579-4836

Email: bvkanna@aol.com

**Additional file 2**
File format: MS Word
Title: Research elective plan
Description: A detailed timetable of daily activities to guide the resident through the different days and components of the rotation

| **Day #** | **Research Elective Component** | **Assignments** | **Deadlines** |
| --- | --- | --- | --- |
| 1 | AM Session:  a) Introduction to elective  PM Session:  a) Discuss specific course work | a) Select topic of interest  b) Required reading (Hypothesis testing: P value & CI)  c) Schedule training session with librarian | a) General selection of area of focus for evidenced based medicine (EBM) and randomized controlled trial (RCT) – Day 2  b) Library on-line resources training – Day 5 |
| 2 | AM Session:  a) Research topics – Hypothesis testing: P value / CI  PM Session:  a) EBM Topic selection | a) Select RCT of interest  b) Required reading (RCT) | a) Selection of RCT – Day 3 |
| 3 | AM Session:  a) Research topics – Critique of RCTs  PM Session:  a) Selection of RCT for critique | a) Designing EBM review  b) Required reading (Study Designs) | a) Selection of specific EBM topic – Day 4 |
| 4 | AM Session:  a) Research topics –Study Designs  PM Session:  a) Formulating EBM topic | a) EBM literature search and RCT write up  b) Required reading (RCTs & Study designs) | a) RCT initial write up – Day 5 |
| 5 | AM Session:  a) Research topics – Review of weeks topics  PM Session:  a) Reviewing RCT progress | a) EBM literature search and RCT write up  b) Required reading (RCTs & Study designs; Bayesian statistics) | a) RCT final write up – Day 6 |

| **Day #** | **Research Elective Component** | **Assignments** | **Deadlines** |
| --- | --- | --- | --- |
| 6 | AM Session:  a) Research topics – Bayesian statistics  PM Session:  a) Reviewing RCT write up | a) EBM initial write up  b) Required reading (Summary measures)  c) Research protocol – initial design | a) EBM write up – Day 10  b) Research protocol initial design – Day 10 |
| 7 | AM Session:  a) Research topics – Summary measures  PM Session:  a) Reviewing EBM write up | a) EBM write up  b) Required reading  (review of RCT design)  c) Research protocol – initial design | Same as above |
| 8 | AM Session:  a) Research topics –  Reviewing statistics  PM Session:  a) Reviewing EBM write up | a) EBM write up  b) Research protocol  c) Required readings review | Same as above |
| 9 | AM Session:  a) Research topics – Summary measures  PM Session:  a) Reviewing EBM write up | a) EBM write up  b) Research protocol  c) Required readings review | Same as above |
| 10 | AM Session:  a) EBM write up  b) Research protocol write -up  PM Session:  Research rotation test | None | a) Research rotation test  b) Submit EBM topic  c) Complete evaluation of research rotation  d) Submit research rotation data sheet  e) Research elective evaluation and feed back |
