# Supplementary material for: The research rotation: competency-based structured and novel approach to research training of internal medicine residents
Source: BMC Med Educ. 2006 Oct 17;6:52. doi: 10.1186/1472-6920-6-52 (PMC1630691; doi:10.1186/1472-6920-6-52)
Supplement: Additional File 3 — Research rotation competency based evaluation form. An ACGME competency based evaluation provided at the end of the rotation to residents based on performance of the resident among the various research rotation components [file 1472-6920-6-52-S3.doc]

# Title:

# The research rotation: competency-based structured and novel approach to research training of internal medicine residents

**Authors:**

Balavenkatesh Kanna^1^ Associate Program Director of Internal Medicine

Assistant Clinical Professor of Medicine

Changchun Deng ^2^

Savil N.Erickson ^3^

Jose A. Valerio^4^

Vihren Dimitrov^5^

Associate Program Director of Internal Medicine

Assistant Clinical Professor of Medicine

Anita Soni^6^

Chair & Program Director of Internal Medicine

Associate Professor of Medicine

**Institutional Affiliation:**

^1,2,5,6^ Department of Internal Medicine, Lincoln Medical & Mental Health Center, New York USA, Affiliated with Weill Medical College of Cornell University, NewYork USA

^3,4^ Research Assistant Program of the Graduate Medical Education Office, Lincoln Medical & Mental Health Center, New York USA, Affiliated with Weill Medical College of Cornell University, NewYork USA

**Corresponding Author:**

Name: Balavenkatesh Kanna MD MPH
Address: 500, Central Park Avenue, Unit # 437, Scarsdale NewYork 10583

Phone: 914-912-8320

Office: 718-579-5000 ext 5016

Fax: 718-579-4836

Email: bvkanna@aol.com

**Additional files 3**
File format: MS Word
Title: Research rotation competency based evaluation form
Description: An ACGME competency based evaluation provided at the end of the rotation to residents based on performance of the resident among the various research rotation components

PGY-1 **X**  PGY-2 PGY-3 PGY-4______

Resident’s Name: Rotation Name: Research Elective

Attending’s Name: Rotation Period: Evaluation Date:

In evaluating the resident’s performance, use as your standard the level of knowledge, skills and attitudes expected from the clearly satisfactory resident at this stage of training. **For any component that needs attention or is rated a 4 or less, please provide specific comments and recommendations on the back of the form.**  Be as specific as possible, including reports of critical incidents and/or outstanding performance. Global adjectives or remarks, such as “good resident,” do not provide meaningful feedback to the resident.

# RESEARCH ELECTIVE SPECIFIC EVALUATION

| **1. Evidenced based topic data gathering (Practice-based learning competency)**  Unable to utilize available resources  to access, review, collect and analyze  medical literature essential for specific research question or practice problem, unable to summarize findings and present in a clear manner | **Unsatisfactory Satisfactory Superior**  **1 2 3 4 5 6 7 8 9**  **** Performance needs attention  **** Insufficient contact to judge | Able to utilize available resources  to access, review, collect and analyze  medical literature essential for specific research question or practice problem,  able to summarize findings and present in a clear manner |
| --- | --- | --- |
| **2. Designing research project (Medical knowledge & Interpersonal skills competency)**  Limited knowledge of basic skills required for study design, methods and analysis; minimal interest in learning; does not understand or comprehend research methodology, interacts and communicates poorly with allied health professionals in project design and methods | **1 2 3 4 5 6 7 8 9**  **** Performance needs attention  **** Insufficient contact to judge | Exceptional knowledge of basic research skills; highly resourceful development of knowledge; comprehensive understanding of research methodology, interacts and communicates well with allied health professionals in project design and methods |
| **3. Performance improvement measures - data collection & analysis (System based Learning competency)**  Disinterested in collecting, databasing and analyzing information relevant to performance improvement. Fails to understand the importance of the analysis of practice data in improving patient care | **1 2 3 4 5 6 7 8 9**  **** Performance needs attention  **** Insufficient contact to judge | Very interested in collecting, databasing and analyzing information relevant to performance improvement. Understand the importance of the analysis of practice data in improving patient care and enthusiastically participated in the data collection and analysis |
| **4. Critique of medical literature (Medical Knowledge competency)**  Unable to understand objectives, study designs, methods and basic statistical analytical methods used in research studies and interpret the results with systematic critique of the various components in a research study | **1 2 3 4 5 6 7 8 9**  **** Performance needs attention  **** Insufficient contact to judge | Able to understand objectives, study designs, methods and basic statistical analytical methods used in research studies and interpret the results with systematic critique of the various components in a research study in an excellent manner. |
| **5. Research Knowledge and Interest**  **(Medical knowledge & Professionalism competencies)**  Exhibits poor interest in acquiring basic research skills, does not follow reading instructions provided and failed to develop basic understanding regarding the scope of skills and knowledge required for research, participated in learning the course objectives with enthusiasm, responsibility and timeliness | **1 2 3 4 5 6 7 8 9**  **** Performance needs attention  ****Insufficient contact to judge | Exhibits great interest in acquiring basic research skills while following reading instructions provided and has develop a sound basic understanding of the scope of skills and knowledge required for research, participated in learning the course objectives with enthusiasm, responsibility and timeliness |

**Resident's Overall Clinical Competence 1 2 3 4 5 6 7 8 9**

**in Internal Medicine on Rotation** ****Performance needs attention

**Instructor's Comments:**

**Signatures: Resident's ________________________________Instructor's_______________________________**
